# Parametrical Optomechanical Oscillations in PhoXonic Whispering Gallery Mode Resonators

**DOI:** 10.1038/s41598-019-43271-x

**Published:** 2019-05-09

**Authors:** Xavier Roselló-Mechó, Daniele Farnesi, Gabriele Frigenti, Andrea Barucci, Alberto Fernández-Bienes, Tupak García-Fernández, Fulvio Ratto, Martina Delgado-Pinar, Miguel V. Andrés, Gualtiero Nunzi Conti, Silvia Soria

**Affiliations:** 1 Department of Applied Physics and Electromagnetism-ICMUV, University of Valencia, Burjassot, Spain; 2 CNR-IFAC Institute of Applied Physics “N. Carrara”, 50019 Sesto Fiorentino, Italy; 3 Centro Fermi - Museo Storico della Fisica e Centro Studi e Ricerche “Enrico Fermi”, Compendio del Viminale, Piazza del Viminale 1, 00184 Rome, Italy; 4 LENS-UniFi European Laboratory Nonlinear Spectroscopy- Università degli studi Firenze, 50019 Sesto Fiorentino, Italy; 5 UNAM, Universidad Nacional Autónoma de México, Mexico City, Mexico; 6 Universidad Autónoma de la Ciudad de México (UACM), Mexico City, Mexico

**Keywords:** Optics and photonics, Other photonics

## Abstract

We report on the experimental and theoretical analysis of parametrical optomechanical oscillations in hollow spherical phoxonic whispering gallery mode resonators due to radiation pressure. The optically excited acoustic eigenmodes of the phoxonic cavity oscillate regeneratively leading to parametric oscillation instabilities.

## Introduction

Whispering gallery mode resonators (WGMR) have attracted a great interest in the last decades. WGMR have been fabricated in different geometries, solid and hollow, spherical, toroidal, and bottle-shaped^1^. Hollow spherical WGMR or microbubble resonators (MBR) are the newest members of this family of resonators. The approach used for their fabrication is based on surface tension driven plastic deformation on a pressurized capillary^2,3^, similar to glassblowing. Using such technique we are able to fabricate large surface area and thin spherical shells with high quality factor (Q).

MBR are efficient phoxonic cavities that can sustain both photons and phonons, either optical or acoustic^4,5^. It has been demonstrated that MBR can be used to study Kerr modulation and Stimulated Brillouin Scattering (SBS)^5–7^. The acoustic phonons responsible for SBS are related to the material and fall in the GHz range for bulk silica. Radiation pressure is another mechanism that also leads to excitation of acoustic phonons at lower frequencies, in the range of hundreds of kHz to tens of MHz in the case of silica MBR^8^. The oscillations are regenerative, with a threshold behavior and without external modulation of the pump wave. Their frequencies are the mechanical eigenfrequencies of the cavity^9–11^.

The effects of radiation pressure have been studied in different geometrical WGMR: microspheres^9^, toroids^10^ and solid bottles^11^. This effect is due to the motion of the interfaces, whereas in the case of very thick hollow bottles bulk electrostriction plays a fundamental role, exhibiting acoustic phonons with high frequencies, up to tens of GHz in silica^4,12^. SBS depends on the resonator host material and relates to bulk acoustic modes. In the case of radiation pressure, the acoustic waves are surface waves with an efficient transfer of the optical energy into the mechanical modes. Thin walled MBR exhibit strong optomechanical effects like toroids due to their low stiffness and very dense spectral characteristics^6,13^. Such properties made MBR very attractive for optomechanical interactions, even for larger devices.

The increasing interest in optomechanical interactions is due not only to a fundamental fascination but also to a broad range of possible applications^14^. Among these applications can be included small force measurements, mass and displacement, gravitational wave detection, creation of non-classical states of light. The MBR used in this study are thin spherical shells exhibiting parametrical optomechanical oscillations at low excitation threshold that can coexist with nonlinear optical frequency generation such as SBS, four wave mixing (FWM) and frequency combs. Their behavior is very similar to that of toroidal WGMR^15^: nonlinear phenomena were observed when the cavity was vibrating. The resonant mode is thermally self-locked to the pump laser by tuning the laser from high to low frequencies^16^. However, we also observed that for certain locking conditions (high pump power) the mechanical oscillation was amplified and nonlinear phenomena were suppressed^17^. In this case, in agreement with Braginsky theory^18^, we observed mechanical oscillations of only one mechanical mode and the optomechanical parametrical oscillations (OMPO) continue as long as the CW pump power is maintained.

Summarizing, we report the observation of parametrical optomechanical oscillations, coexistence of OMPO and nonlinear phenomena, suppression of OMPO, and a detailed modeling of the mechanical oscillations, showing also very dense mechanical mode spectra.

## Experimental Set Up

### Microbubble fabrication

**Figure 1:**
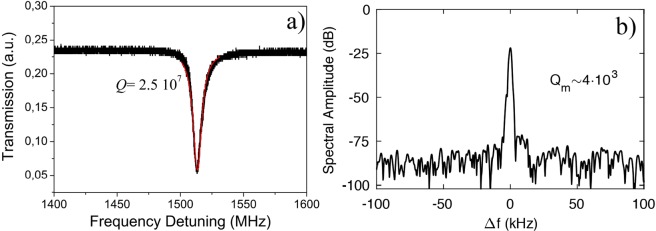
(**a**) Optical Q factor (2.5 × 10^7^), pump wavelength of 1550 nm and pump power of 0.5 mW and (**b**) Mechanical Q factor (4 × 10^3^) for a MBR of a diameter ~460 μm and wall thickness of ~5.5 μm, measured at pump wavelength of 1550 nm and pump power of 80 mW.

The MBR were fabricated using a technique similar to glassblowing, based on surface tension driven plastic deformation on a pressurized capillary. The fabrication technique has been described in detail in a previous paper^3^. The MBR were fabricated using a capillary with an internal diameter of 200 μm and external diameter of about 280 μm (Postnova Z-FSS-200280 capillary). The microbubble diameters used in these experiments span from 420 μm up to 780 μm with wall thicknesses from 2 to 6 μm ± 0.5 μm. The capillary was not always tapered before inflation; thus, some bubbles were thicker compared to our previous works^5,6^. Figure 1 shows a representative optical quality factor Q that is over 10^7^ whereas the mechanical quality factor is in excess of 10^3^.

### Experimental setup

**Figure 2:**
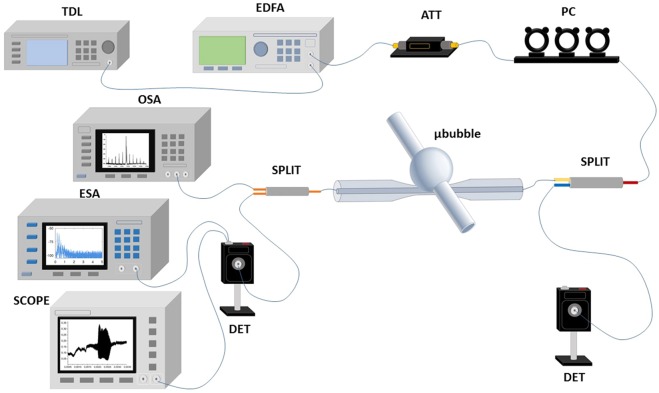
Experimental set-up.

Figure. 2 shows the experimental setup. An erbium-doped fiber amplifier (EDFA) is used for amplifying the laser emission from a tunable diode laser (TDL). An attenuator (ATT) and a polarization controller (PC) are used for power and polarization control before coupling the pump laser into the MBR by means of a homemade tapered fiber. Part of the signal is sent into an optical spectrum analyzer (OSA, ANDO AQ6317B) and part into a photo-detector (DET) and oscilloscope (TEKTRONIX, DPO7104) by means of splitters. The optomechanical oscillations were also characterized by monitoring the output of the PD with an electrical spectrum analyzer (ESA, Rhode Schwarz FSL 9 kHz-6 GHz). In all measurements, the tapered fiber was kept in contact with the MBR in order to minimize the fluctuations of the coupling condition. The generated frequencies were detected on an OSA directly transmitted by the tapered fiber. Numerical simulations were performed using a 3D finite element method (FEM) in COMSOL Multiphysics.

## Results and Discussion

MBR are spheroidal WGMR with dense optical and mechanical spectra. Thus, their geometrical characteristics can explain the observed phenomenology. The geometrical dispersion is normal and large, whereas the material dispersion is anomalous and larger than the geometrical one at the wavelengths under study. The total dispersion is anomalous, also with values in the order of several hundreds of kHz, as expected for very large MBR^6^. Thus, Kerr comb formation is allowed for all MBR used in this work. The diameters of the MBR have been chosen based on their dispersion^6^.

We performed numerical simulations using FEM in COMSOL Multiphysics, in order to calculate the mechanical eigenmodes of microbubbles within a range of geometrical parameters covering all our MBR. A geometrical representation as faithful as possible in terms of radii, thicknesses and fillets was designed by a CAD module available in COMSOL. Some geometrical parameters as the length of the capillary holding the MBR and the radius of the MBR were varied in order to understand their impact on the observed mechanical spectra. As mentioned before, MBR are spheroidal resonators with a non-uniform thickness, specifically with the wall being thinnest at the equator. A prolate spheroid defines the inner surface of the MBR. Compared to solid WGMR, MBR are lighter and so the frequencies of the mechanical oscillations are lower.

**Figure 3:**
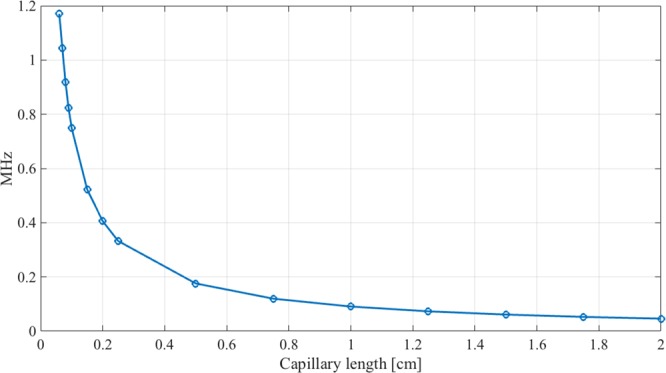
COMSOL Multiphysics calculated influence of the capillary length on the frequency of the fundamental breathing mode for a MBR with a diameter of 500 μm and wall thickness of about 5 μm.

MBR also show a complex spectrum of mechanical modes. Ideally, these modes belong to different families. Radial breathing modes (m = 0) hold cylindrical symmetry corresponding to the extension-compression of the spherical shell across the longitudinal axis and are non-degenerate. Rocking modes (m = 1) correspond to a lateral bending of the MBR displaying double degeneracy with a mutual orientation of 90°. Wineglass modes (m > 1) are more balanced vibrations exhibiting more nodes and occasionally a sharp localization along the equatorial circumference of the MBR and maintaining double degeneracy at angles that depend on the order. However, most modes generated by the numerical solution exhibit an intricate shape that is hardly attributable to any major class. Figure 3 shows the influence of the capillary length on the frequency of the fundamental breathing mode for a MBR with a diameter of 500 µm and a wall thickness of 5 µm and shows the occurrence of mechanical vibrations that can reach down to a regime of a few tens kHz. With respect to previous papers that neglected the role of this component and presented fundamental breathing modes with frequencies around 1 MHz^8,11^, we posit that a description of these vibrations as longitudinal modes in a massive capillary hosting a light microbubble may be crucial.

**Figure 4:**
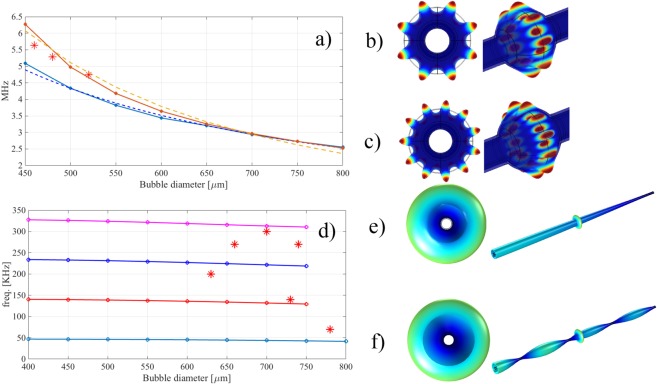
COMSOL Multiphysics numerical simulations and experimental data. Effect of the diameter of the MBR on the excitation of the mechanical oscillations for (**a**) higher frequency modes displaying 11 (dark red full line and dots) or 8 nodes (blue), relevant fitting (dashed lines) and experimental data (stars), (**b**) and (**c**) shape of wine glass or hybrid modes with 8 (**b**) or 11 (**c**) nodes, (**d**) lowest frequency breathing modes with 1 node (light blue full line and dots), 2 nodes (red), 3 nodes (blue) and 4 nodes (magenta) and experimental data (stars), (**e**) and (**f**) shape of breathing mode and capillary mode with 1 (**e**) or 4 (**f**) nodes.

Figure 4 displays the effect of the diameter of the microbubble on a representative selection of mechanical modes. We warn the reader that following the same modes over different diameters is tentative, because it is the overall shape of the device that changes with the diameter of the microbubble, i.e. as the diameter of the microbubble grows, the diameter and wall thickness of the capillary remains the same and the equatorial wall thickness of the microbubble decreases according to ${t}_{microbubble}\cong {t}_{capillary}\times {(\frac{{d}_{capillary}}{{d}_{microbubble}})}^{2}$^19^. In particular, we tracked a few lowest energy breathing modes as well as a couple of higher energy modes in a regime of a few MHz displaying wineglass or hybrid profile, which were chosen for their strong localization along the equatorial circumference of the microbubble (in terms of elastic energy or strain, see Fig. S2 and relevant discussion in Supplementary Information) as well as an easily identifiable shape. The frequency of the lowest energy breathing modes scales with the number of nodes in the longitudinal oscillation of the capillary and does not depend much on the diameter of the microbubble. Instead, as a rule of thumb, we found that the natural frequency of the wineglass or hybrid structures around a few MHz approximately scales as ${{d}_{microbubble}}^{-a}$, where *a* typically falls in a range between 1 and 2.

It is worth to mention that in the range of diameters that we have studied, the spectral density of mechanical modes is very high, i.e. on average about 1 mode every 5 kHz until 5 MHz, for a MBR with a diameter of 500 µm (see supplementary information, Fig. S1). The excitation of a certain mode depends on the coupling conditions, the optical resonance that is thermally locked and the overlap of the optical and mechanical modes. In this context, a direct correlation between simulations and experimental modes is hardly possible, due to the complexity and extreme density of the mechanical spectrum. However, we have identified two different ranges of frequencies, a low frequency one for submillimetric MBR (70–700 kHz) and a high frequency one for MBR with diameters smaller than 600 µm (1–60 MHz). The low frequency range must be dominated by the effect of the capillary. We hypothesize that these oscillations may correspond to lowest energy longitudinal modes in the capillary and the one that resonates also depends on the exact location of the MBR along the capillary with respect to those of its nodes. The high frequency range seems to follow a scaling behavior compatible with the evolution of a single higher energy wineglass or hybrid mode. The reason why certain MBR resonate at lower or higher frequency will be the matter of future research.

**Figure 5:**
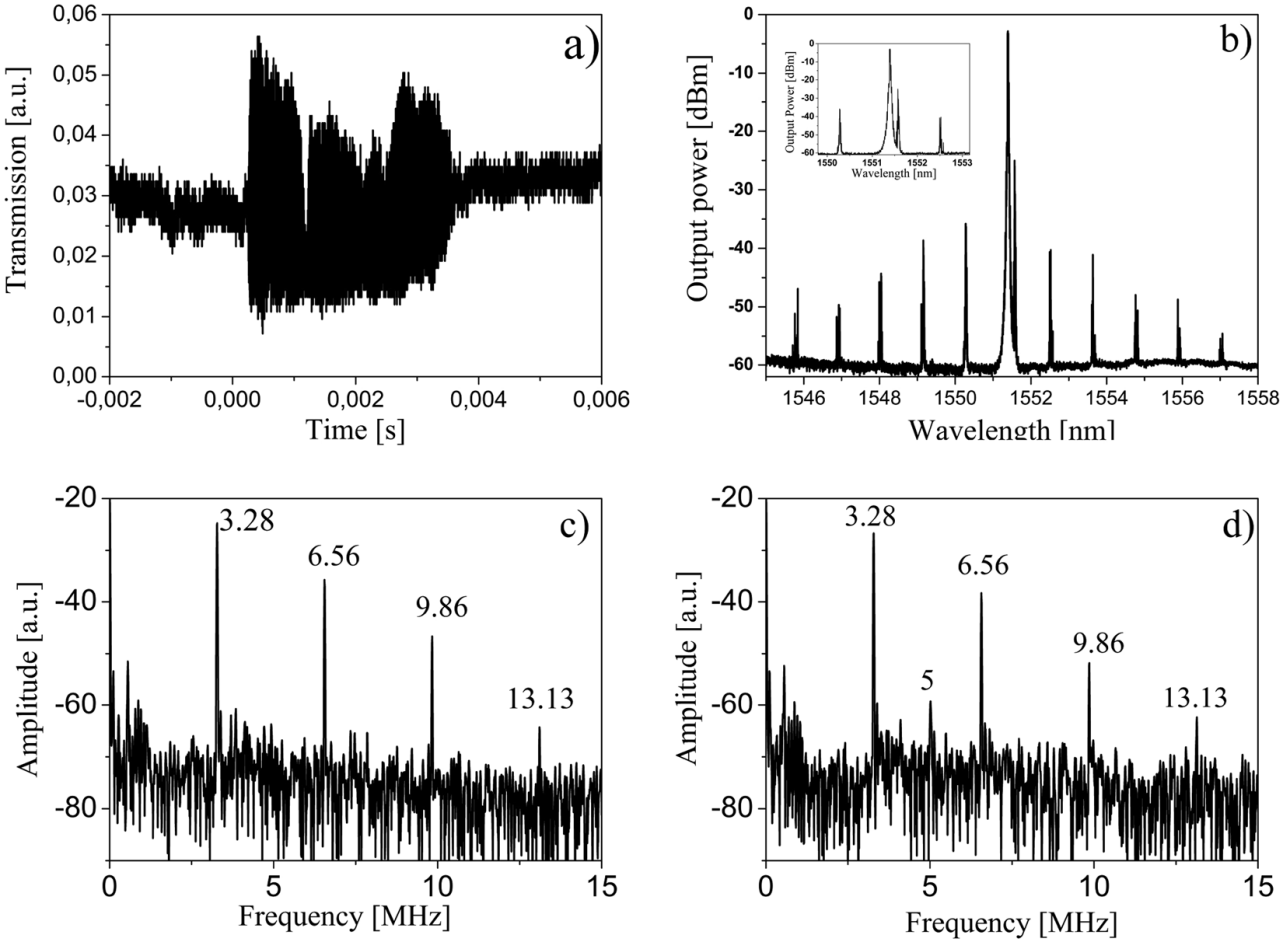
(**a**) Typical transmission spectra obtained at 72 mW launched pump power above the mechanical parametrical oscillation threshold at 1551.344 nm pump wavelength; (**b**) native Kerr comb and second order SBS in forward direction: pump power 72 mW, pump wavelength 1551.344 nm (inset: close up of the spectrum showing the 2^nd^ order SBS laser line and the first pair of FWM lines); (**c**) and (**d**) experimental oscillations at 72 mW and 1553.476 nm for one single family (**c**) or two different families (**d**) of excited modes.

Figure 5a shows the transmission when scanning the pump laser from short to long wavelengths around the resonant wavelength of 1551.344 nm for a pump power of 72 mW. The diameter of the MBR was about 475 μm and its equatorial wall thickness was about 4 ± 0.5 μm. This particular MBR is thinner than some others used in this work, because the capillary was slightly tapered before inflation. The free spectral range (FSR) of our MBR is about 141 GHz and its quality factor Q exceeds 10^7^. Figure 5b shows the optical hyper-parametrical oscillation acquired in the forward direction displaying a comb with one FSR equidistant sideband. In this particular case, we have realized a “Type I” comb^20,21^. As expected in the forward direction, we have observed the second order SBS Stokes line, separated by 0.18 nm (22 GHz at telecom wavelengths) from the pump. These nonlinear phenomena were observed only when the cavity was vibrating. MBR are spheroidal WGMR with two mode families that are nearly equidistant with two different FSR, namely the azimuthal FSR (described by quantum number l) and the vertical FSR (described by quantum number p)^6,22^. It has been demonstrated that mode families with the same azimuthal but different vertical quantum number overlap spatially and interact among them. The result of such interaction is different frequency spacing and an asymmetry in the optical spectrum^6^. We assume that they raise also different mechanical mode families. Figure 5c and d show the mechanical oscillations at 72 mW of launched pump power for two different coupling conditions at 1553,476 nm, namely coupling at the equator of the MBR (c) or by laterally shifting the taper along the MBR axis (d). The first sharp peak corresponds to a mode at 3.28 MHz together with its harmonics, where only one mechanical mode is excited, in agreement with Braginsky theory^18^. When two or more modes are excited (Fig. 5d), we observed the mechanical mode at 3.28 MHz and up to its 3^rd^ harmonic, together with a mode at about 5 MHz and another one at 552 kHz. As mentioned above, it is quite difficult to completely assign the experimental modes. However, in theory, the modes around 3 MHz belong to the breathing mode family, the modes around 5 MHz belong to the wine glass family, whereas the structural modes in the kHz range involve the whole structure.

When the pump has a higher frequency than the resonance (blue detuning), the cavity resonance supresses the blue sideband and enhances the red one^11,17^ resulting in an amplification of the OMPO. We are always scanning the pump from high to low frequency in order to thermally lock the resonances^16^. Mechanical oscillations are always amplified when the light is coupled to the resonance with blue detuning. Coexistence of OMPO and Kerr combs is possible due to the large MBR dispersion. Measurements shown in Fig. 5a and b were performed when thermal locking was observed first and then coupling was optimised until the nonlinear optical phenomena arose.

**Figure 6:**
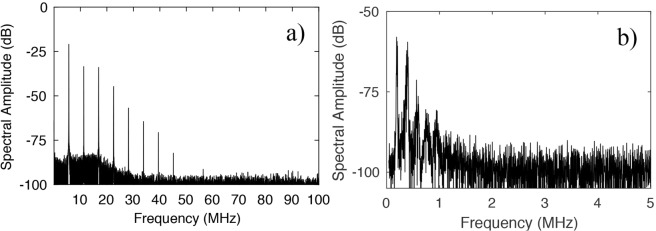
Mechanical oscillations measured at the ESA and oscilloscope: first excited mechanical mode and its harmonics for a bubble of (**a**) 460 µm of diameter at 200 mW and (**b**) 630 µm of diameter at 150 mW for a pump wavelength of 1550 nm.

We repeated the measurements by increasing the pump power and optimising the detuning till the resonances were locked to a stable warm equilibrium^16^ with an MBR with a diameter of about 460 μm and a thickness of about 5.5 ± 0.5 μm. In this case, the MBR was fabricated without tapering the capillary before glassblowing. The launched pump power was about 200 mW. RF signals appeared at the ESA in multiples of 5.6 MHz. The oscillation frequency is compatible with a wine glass mode and neither comb lines nor SBS was generated, despite the dispersion was very large and anomalous. The decrease of FWM efficiency is due to the different coupling conditions^6^. Figure 6a shows the mechanical oscillations at 200 mW and coupling close to the equator. The fundamental oscillation corresponds to a wine glass mode at 5.63 MHz, while the other spectral lines are its higher harmonics. In this case, high pump powers can excite higher order harmonics. We observed up to as many as seven harmonics. As shown in Fig. 4, the frequency of the oscillation decreases with the increase of the diameter of the MBR, in line with our FEM simulations. Figure 6b shows the frequency spectrum of the OMPO of a MBR of diameter about 630 μm and thickness below 3 μm. The first frequency that we measured was about 200 kHz and we observed up to four harmonics for a launched pump power of 150 mW.

The peak spectral amplitude versus the optical pump power shows a laser threshold behavior for the fundamental mode, with gain saturation at about 140 mW of launched pump power. We have not calculated the theoretical threshold power because we cannot know the frequency detuning between the pump and the resonant wavelength. This is a direct consequence of using the thermal locking method. We performed the measurements in two different conditions: a) at the detuning when thermal locking was observed for the first time and b) when the OMPO fully suppressed the nonlinear phenomena. In both cases, we are always on the blue side of the optimal detuning^11,17^. The narrowing of the linewidth is also an indication of the laser-like behavior of the MBR. The phonon laser behavior can be observed in Figs S2 and S3 in the supplementary materials.

**Figure 7:**
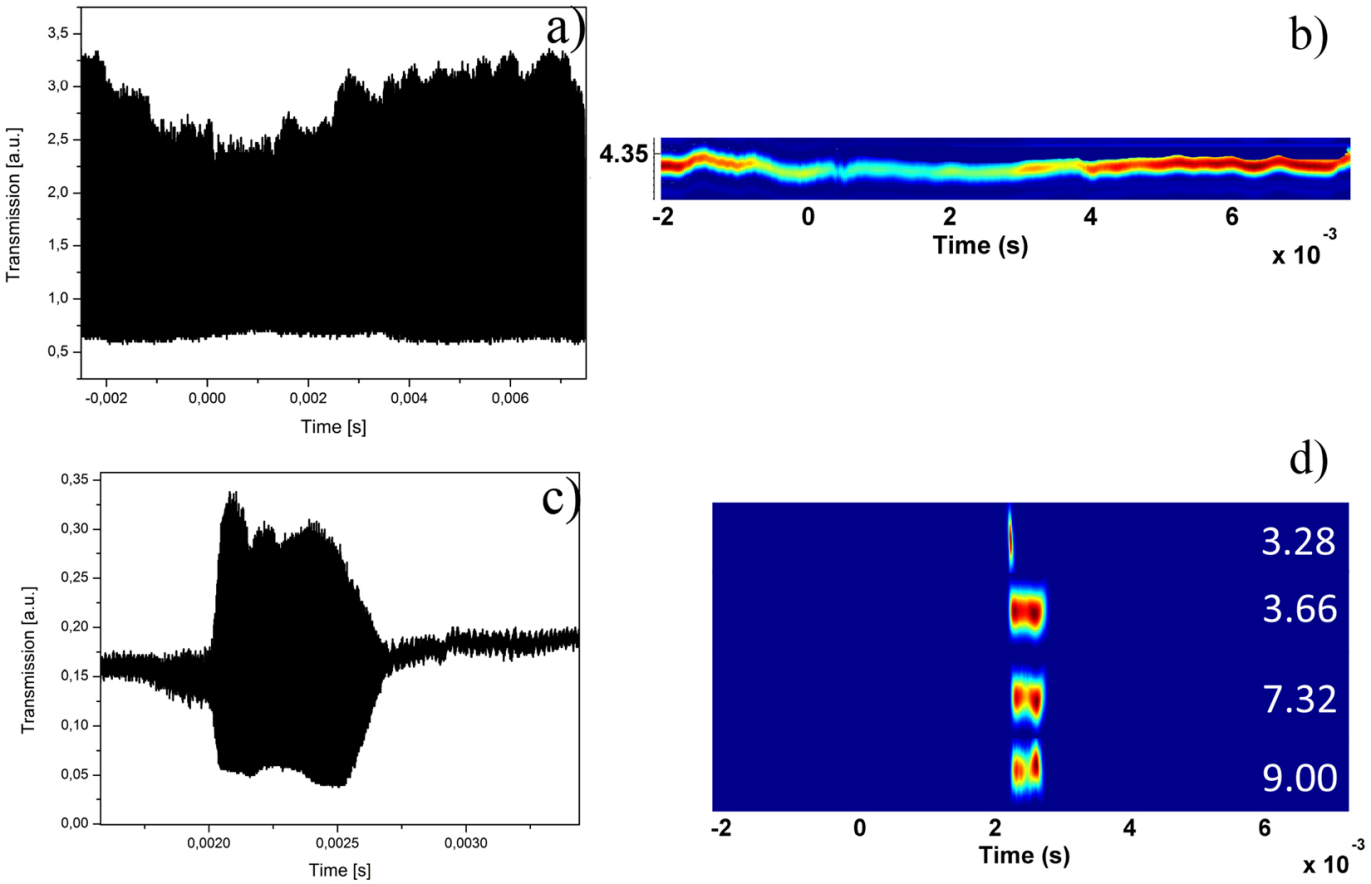
Oscillations for two different launched pump power for a MBR of about 480 µm of diameter at 1544.54 nm: (**a**) and (**b**) pump power 200 mW in time domain (**a**) and frequency domain (**b**),(**c**) and (**d**) pump power 72 mW in time domain (**c**) and frequency domain (**d**). The units of the amplitude in the frequency domain are arbitrary.

We checked the thermal drift of the mechanical resonance and the temporal stability by increasing and decreasing the launched pump power. As expected, there is a small shift of about 20 kHz due to thermal drift induced at high pump powers (see Supplementary Materials, Fig. S4). Spectral densities and OMPO details can be seen in the Supplementary Materials, Figs S5, S6 and S7. Figure 7 shows the time-frequency distribution map for two different launched pump powers for the same MBR of about 480 µm of diameter. For high pump powers and locking into a stable warm equilibrium, the oscillations are fully distributed during the scan time of the transmission, with a slight variation in the amplitude of the oscillation. For low pump powers, the mechanical oscillation appears only when the intracavity power is above threshold.

## Conclusions

In conclusion, we report the observation of coupling between optical and mechanical modes in MBR, phonon lasing, and suppression and coexistence of OMPO with nonlinear phenomena, such as Kerr comb formation and SBS. We have modeled the acoustic behavior of MBR by commercial COMSOL Multiphysics. The mode shapes and natural frequencies were confirmed experimentally in close agreement. The spectral density of the mechanical modes is very high, similarly to the optical ones. Depending on the coupling conditions and the locking of the resonance, we observed OMPO of either one mode, according to Braginsky’s theory, or several modes, in agreement with the observation of OMPO in toroids^10^. Besides the fundamental aspect of this study, our results pave the way to a new class of inertial devices, exploiting 3D acoustic waves in symmetric MBR on a microscale.

## Supplementary information

Supporting Information SREP-18-38315A
