# Supplementary material for: Parametrical Optomechanical Oscillations in PhoXonic Whispering Gallery Mode Resonators
Source: Sci Rep. 2019 May 9;9:7163. doi: 10.1038/s41598-019-43271-x (PMC6509163; doi:10.1038/s41598-019-43271-x)
Supplement: Supplementary file 1 — Supporting Information SREP-18-38315A [file 41598_2019_43271_MOESM1_ESM.pdf]

# Supporting Information

# Parametrical Optomechanical Oscillations in PhoXonic Whispering Gallery Mode Resonators

Xavier Rosello Mecho[1], Daniele Farnesi[2], Gabriele Frigenti[2,3,4], Andrea Barucci[2], Alberto Fernández-Bienes[5], Tupak Garcia- Fernández[6], Fulvio Ratto[2], Martina Delgado Pinar[1], Miguel V. Andrés[1], Gualtiero Nunzi Conti[2,4], Silvia Soria[2*]

[1]Dep. of Applied Physics and Electromagnetism-ICMUV, University of Valencia, Burjassot, Spain
[2]CNR-IFAC Institute of Applied Physics "N. Carrara", 50019 Sesto Fiorentino, Italy
[3]LENS-UniFi European Laboratory Nonlinear Spectroscopy- Università degli studi Firenze, 50019 Sesto Fiorentino, Italy
*[4]Centro Studi e Ricerche "E. Fermi", P. Viminale 2, 00184 Rome, Italy*
[5]UNAM, Universidad Nacional Autónoma de México, Mexico City, Mexico
[6] Universidad Autónoma de la Ciudad de México (UACM), Mexico City, Mexico
*Correspondence to s.soria@ifac.cnr.it

## 1. Mechanical modes from numerical simulation

Figure S1 shows the spectral density of the mechanical modes within the frequency range of 0-6 MHz calculated by COMSOL Multiphysics.

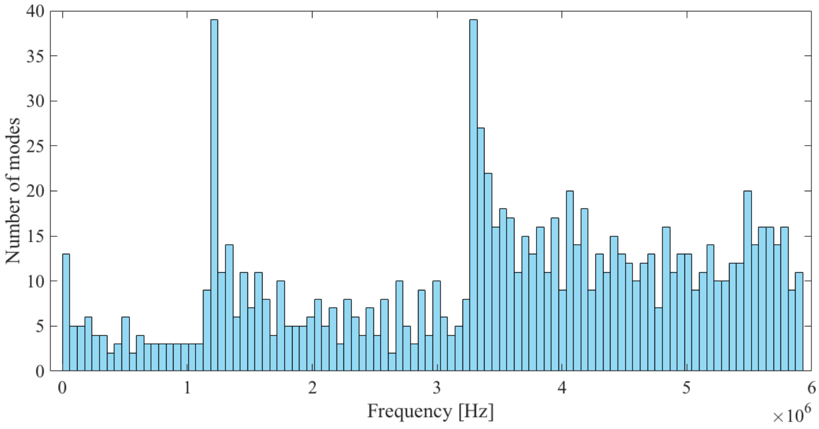


Fig. S1. Spectral density for a MBR of a diameter of about 500 um showing 1 mode every 5 kHz (binning=60 kHz

## 2. Phonon Laser

Figure S2 shows the spectral amplitude versus the launched pump power. The plot reveals a laser threshold behaviour for the fundamental mode at 5.63 MHz for a MBR of a diameter of about 460 um. In addition to the threshold behaviour we also observed gain saturation about 120 mW of launched pump power. Figure S3 the narrowing of the laser line and the shifting of the line when increasing the launched pump power.

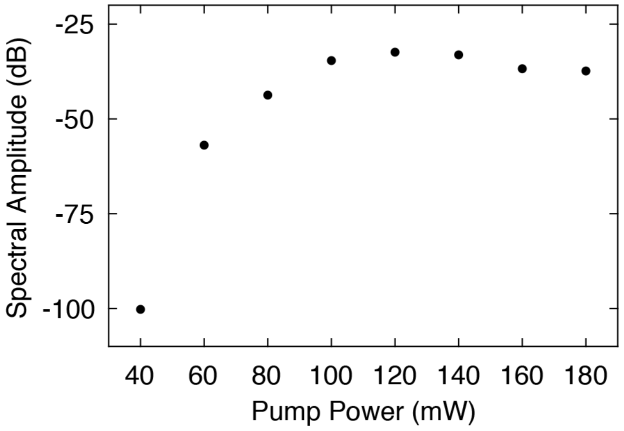


Fig. S2. Phonon lasing in a MBR of 460 um of diameter. Threshold behaviour and gain saturation are clearly seen.

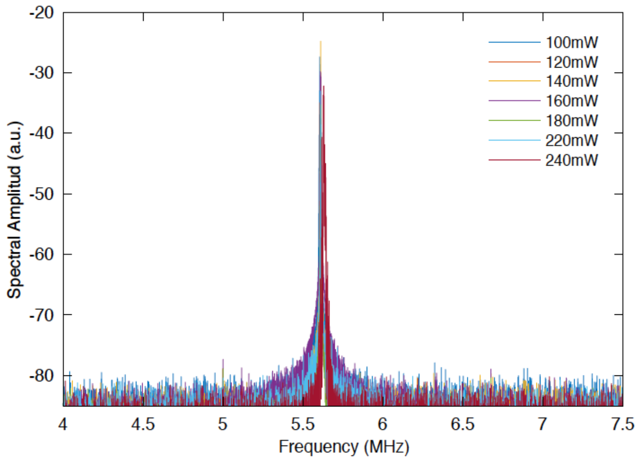


Fig. S3. Peak Spectral Amplitude for different pump powers showing the expected narrowing of the linewidth.

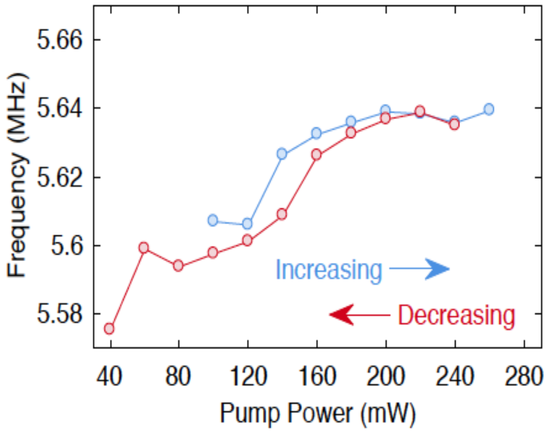


Fig. S4. Frequency shift for different pump powers.

## 3. Optical Resonances

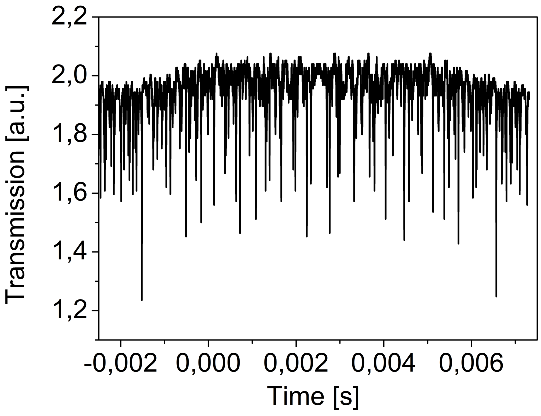


Fig. S5. Optical Spectral density for a MBR of a diameter of about 500 um below the optomechanical oscillation threshold.

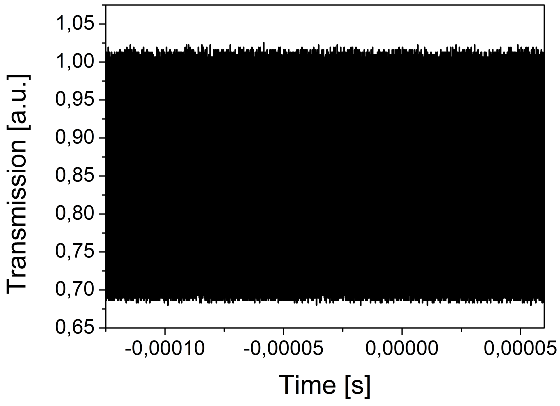

Fig. S6. Optomechanical parametric oscillation for a launched pump power of about 200 mW.

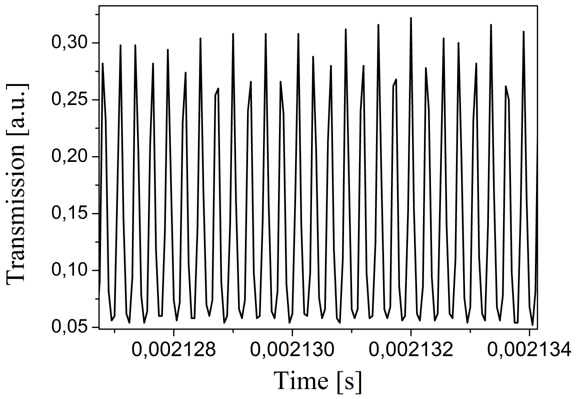


Fig. S7. Detail of an optomechanical parametric oscillation for a launched pump power of about 72 mW at a pump wavelength of 1551.344 of a MBR of about 480 um.
